# Shikonin alleviates doxorubicin-induced cardiotoxicity via Mst1/Nrf2 pathway in mice

**DOI:** 10.1038/s41598-024-51675-7

**Published:** 2024-01-09

**Authors:** Hu Tuo, Wenjing Li, Wei Zhao, Juan Zhao, Danni Li, Lin Jin

**Affiliations:** 1 Department of Pediatrics, Renmin Hospital of Wuhan University, Wuhan, China; 2 Department of Anesthesiology, Renmin Hospital of Wuhan University, Wuhan, China; 3 Department of Orthopedics, Renmin Hospital of Wuhan University, Jiefang Road 238, Wuhan, 430060 China

**Keywords:** Cardiovascular diseases, Cell signalling

## Abstract

Doxorubicin (DOX) is a popular and potent anticancer drug, but its cardiotoxicity limits its clinical application. Shikonin has a wide range of biological functions, including antioxidant and anti-inflammatory effects. The aim of this study was to investigate the effects of shikonin on DOX-induced cardiac injury and to identify the underlying mechanisms. Mice receiving shikonin showed reduced cardiac injury response and enhanced cardiac function after DOX administration. Shikonin significantly attenuated DOX-induced oxidative damage, inflammation accumulation and cardiomyocyte apoptosis. Shikonin protects against DOX-induced cardiac injury by inhibiting Mammalian sterile 20-like kinase 1 (Mst1) and oxidative stress and activating the nuclear factor erythroid 2-related factor 2 (Nrf2) pathway. In conclusion, shikonin alleviates DOX-induced cardiotoxicity by inhibiting Mst1 and activating Nrf2. Shikonin may be used to treat DOX-induced cardiac injury.

## Introduction

Doxorubicin (DOX) is an anthracycline antibiotic with anti-tumor properties that can be used to treat a number of malignancies^1^. However, the clinical use of doxorubicin is restricted because of its harmful side effects. The most notable of these is cardiotoxicity, which can occur suddenly or develop over time as dilated cardiomyopathy, abrupt left ventricular dysfunction, or heart failure^2^. Cardiotoxicity caused by DOX is caused by a complicated, multifaceted mechanism. A significant contributor to DOX-induced cell damage, heart dysfunction, and alterations in cellular ultrastructure and energy metabolism is oxidative stress^3–5^. Although DOX-induced cardiotoxicity includes a variety of cellular and molecular pathways, cell death, including apoptosis and necrosis, is ultimately the result^6,7^.

Several cellular mechanisms and signaling pathways have been implicated in cardiac injury, including oxidative stress and inflammatory responses. Among the key signaling pathways involved in the regulation of oxidative stress and inflammation, Mst1 (mammalian sterile 20-like kinase 1) and Nrf2 (nuclear factor erythroid 2-related factor 2) have emerged as key regulators of cardiac injury^8^. Mst1 is a member of the serine/threonine protein kinase family that has been implicated in apoptosis in cardiac cells. Studies have shown that DOX can activate Mst1, leading to cardiomyocyte apoptosis. Thus, Mst1 may be an important mechanism underlying DOX-induced cardiotoxicity. Nrf2, encoded by the NFE2L2 gene, serves as a pivotal transcription factor orchestrating the cellular response to stress by regulating various antioxidant and detoxification genes, thereby safeguarding cardiac cells against oxidative stress and toxic insults^9^. Studies have shown that DOX can inhibit Nrf2 activity, leading to cardiomyocyte toxicity and apoptosis. Therefore, Nrf2 may be a critical target in DOX-induced cardiotoxicity. In addition, many natural products and compounds can also prevent and treat DOX cardiotoxicity by activating Nrf2 signaling pathway^10^. To lessen DOX-induced cardiotoxicity, it is crucial to develop safe and efficient adjuvant treatment techniques.

**Figure 1:**
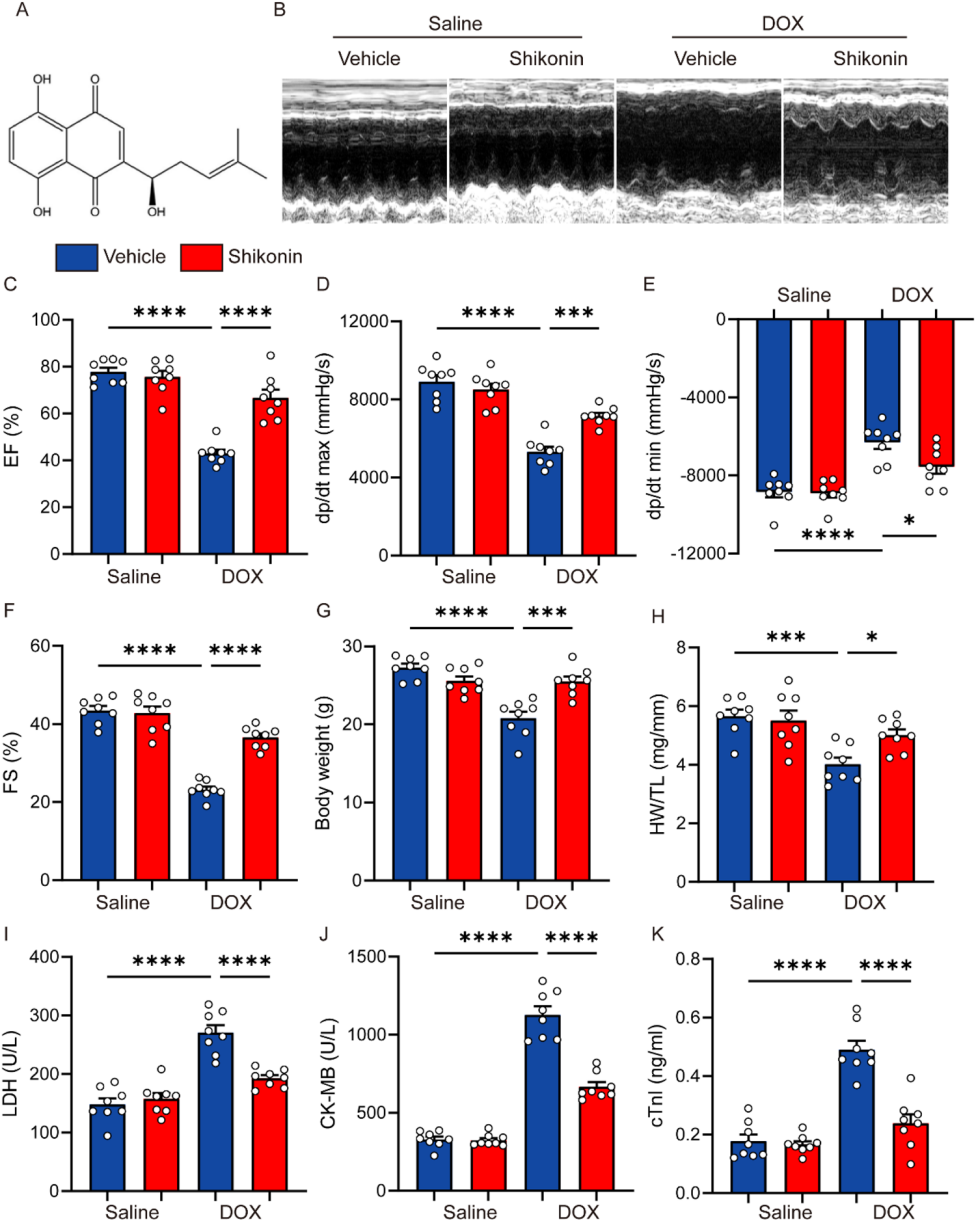
Shikonin improved cardiac function and alleviated cardiac injury in mice with DOX treatment. (**A**) The chemical structure of shikonin was shown. (**B**) Representative echocardiography M-mode images obtained from mice at indicated groups after DOX administration. (**C–F**) Left ventricular ejection fraction (EF%), maximal left ventricular pressure rising rate (dp/dt max), minimal left ventricular pressure rising rate (dp/dt min), left ventricular fraction shortening (FS%) were quantified via echocardiography (n = 8). (**G**) Body weight of four groups (n = 8). (**H**) Statistical results of the heart weight (HW)/tibia length (TL) (n = 8). (**I–K**) Plasma cTnI (cardiac troponin I), creatine kinase (CK) and lactated hydrogenase (LDH) concentrations in mice were measured by ELISA (n = 8). *p < 0.05, ***p < 0.001, ****p < 0.0001, significantly different as indicated.

Shikonin, a naphthoquinone, is a key bioactive substance found in Lithospermum erythrorhizon roots and has potent anticancer properties. As shown in Fig. 1A, the structure of shikonin molecule is 5,8-dihydroxy-2-((1R)-1-hydroxy-4-methyl-3-pentenyl)-1,4-naphthoquinone^11,12^. Shikonin is a very intriguing molecule that has generated a lot of interest in the medicinal chemistry community due to its potential for a wide range of pharmacological effects, including anti-inflammation^13^, anti-cancer^14–17^, cardiovascular protection^18^, anti-microbial^19^, analgesia^20^, anti-obesity^21^, brain protection^22^ and so on. Additionally, recent research showed that shikonin provide protection against cardiovascular illnesses. Shikonin, for instance, reduced sympathetic remodeling in mice with chronic heart failure^23^. Shikonin reduces LPS-induced heart dysfunction by preventing NLRP3 inflammasomes from activating SIRT1-dependently^18^. Cardiomyocyte hypertrophy and fibrosis in Ang II-induced cardiac remodeling in vivo were reduced by Shikonin’s suppression of PKM2^24^. In addition, shikonin ameliorated ISO-induced cardiac injury and dysfunction^25^. Shikonin, a known PKM2 specific inhibitor, treatment of vascular smooth muscle cells resulted in a dose- and time-dependent inhibition of oxLDL-induced proliferation and migration, suggesting that targeting PKM2-dependent glycolysis may open up a new therapeutic path for the treatment of atherosclerosis^26^.

Shikonin’s potential to lessen doxorubicin-induced cardiotoxicity is currently undetermined. We investigated if shikonin can lessen the negative effects of DOX on the heart by combining it with the anti-tumor mechanism and the newly discovered cardiovascular-protective benefits of shikonin. In our investigation, we found that shikonin reduced the oxidative stress, apoptosis, and inflammation caused by DOX while also enhancing heart function.

## Methods

### Animals and treatments

This study was carried out in compliance with the ARRIVE (Animal Research: Reporting of In Vivo Animal Experiments) guidelines. The Institutional Animal Care and Use Committee of Renmin Hospital of Wuhan University (Wuhan, China) authorized all animal experimentation techniques. At Renmin Hospital of Wuhan University, the Animal Care Facility Service kept male C57BL/6J mice (21–24 g; HFK Bioscience, Beijing, China) aged 8 to 9 weeks in a pathogen-free mouse room (12 h of light/12 h of darkness; temperature 22–24 °C). The mice were given unlimited access to water. Under typical laboratory settings of 55.5% relative humidity, 23.2 °C, and 12 h of light, all experimental animals were raised. The ethical committee at Renmin Hospital of Wuhan University accepted this study, and all experimental procedures were carried out in compliance with their institutional policies regarding the care and use of animals (Wuhan, China). Additionally, institutional review board consent was attained. Mice were separated into four groups to study the effects of shikonin on DOX-induced cardiotoxicity (n = 10 per group): Control group; shikonin group; DOX group; and shikonin Plus DOX group. To mimic the cardiotoxicity associated with chronic DOX exposure, mice were intraperitoneally injected with DOX (4 mg/kg) once a week for 3 weeks, or they were given an equal volume of saline as a control. Shikonin dosage (4 mg/kg/day, 25 mg/mL diluted in 0.1% DMSO) or vehicle) was chosen in accordance with a prior study^25^. In control groups, regular saline was administered in place of shikonin (Fig. S1A).

Adenoviral vectors (AdV) were purchased from Hanbio Biotechnology Co. (Shanghai, China) and were used to deliver short hairpin RNA against Nrf2 (shNfe2l2) into 3–5 sites of the left ventricular free wall. In contrast, mice in the control group received an injection of scramble RNA (shRNA). These mice received either an equivalent amount of saline as a control or a dose of DOX (4 mg/kg) 3 days after receiving an AdV injection to mimic the cardiotoxicity that results from chronic DOX exposure.

### Echocardiography and hemodynamics

Echocardiography was performed to evaluate cardiac function at the indicated time points. In brief, echocardiography was performed under continuous anaesthesia with 1.5–2% isoflurane using a Mylab30CV (ESAOTE) ultrasound system with a 15 MHz probe. M-mode tracings derived from the short axis of the LV at the level of the papillary muscles were recorded^27^. In a nutshell, an echocardiogram was done utilizing a Mylab30CV (ESAOTE) ultrasound equipment with a 15 MHz probe while the patient was continuously sedated with 1.5–2% isoflurane. At the level of the papillary muscles, M-mode tracings taken from the short axis of the LV were captured.

Mice were given 2% isoflurane anesthesia, and invasive hemodynamic monitoring was done using a 1.0 F microtip catheter (PVR 1045) attached to a Millar Pressure–Volume System (MPVS-400; Millar Instruments). The PVAN analysis software was used to record and evaluate this data.

At the end of study, the mice were sacrificed with an overdose of sodium pentobarbital (200 mg/kg; i.p.) to harvest the hearts. The heart was quickly excised and rinsed in cold saline solution. The left ventricle was then separated and used for further analysis.

### Neonatal rat cardiomyocytes isolation, culture and transfection

As previously mentioned, Neonatal rat cardiomyocytes (NRCMs) were isolated^28^. Briefly, hearts from Sprague–Dawley rats that were 1 to 2 days old were torn apart and digested in 0.25% trypsin (Gibco) for 10 h at 4 °C. Collagenase continued to break down the heart tissue, separating it into individual cells (Sigma). Cells were suspended in 10% fetal bovine serum (FBS, Gibco) culture media after being centrifuged (800 rpm × 3 min). To remove fibroblasts, the cell suspension was pre-plated twice for 90 min each time. The non-adherent cells were gathered, seeded with high-glucose DMEM with 10% FBS, 100 units/mL penicillin, 100 g/mL streptomycin, and 100 mol/L bromodeoxyuridine (BrdU), and then cultured at 37 °C in a 5% CO_2_ environment. Cells were randomly separated and prepared for additional studies after a 72-h incubation period.

According to the manufacturer’s instructions, siRNA was mixed with Lipofectamine3000 (Thermo Fisher Scientific) in Opti-MEM (Gibco) reduced serum media at room temperature for 15 min for the transfection of Nfe2l2 siRNA (siNfe2l2) and negative control siRNA (ncNfe2l2) (RIBOBIO, Guangzhou, China), The NRCMs were then transfected for 6 h with the appropriate mix according to the experiment’s design. Before continuing with the treatment, the cells were replaced with cell culture media and incubated for 24 h. The siNrf2 groups were designed as follow: (a) ncNfe2l2 group; (b) shikonin + ncNfe2l2 group; (c) ncNfe2l2 group; (d) DOX + ncNfe2l2 + shikonin group; (e) siNfe2l2 group; (f) shikonin + siNfe2l2 group; (g) siNfe2l2 group; (h) DOX + siNfe2l2 + shikonin group. NRCMs were treated with 0.1 μM DOX for 48 h or 0.1 μM shikonin every 8 h according to the experiment design^24^.

### Elisa assay

An ADVIA® 2400 automatic biochemical analyzer (Siemens Ltd.; Tarrytown, NY, USA) was used to detect the levels of cardiac damage markers (Lactate dehydrogenase, LDH; cardiac isoform of tropnin T, cTnT; creatine kinase isoenzymes, CK-MB) and hepatotoxic biomarkers (Alanine transaminase, ALT; aspartate transaminase, AST) in serum.

Using commercially available kits and following the manufacturer’s instructions, the levels of glutathione (GSH), malondialdehyde (MDA), total superoxide dismutase (SOD), and tumor necrosis factor-*α* (TNF-*α*) were assessed The Nanjing Jiancheng Institute of Biotechnology provided the GSH assay kit (#A061), MDA assay kit (A003-1-2), and total SOD activity (A001-3-2) (Nanjing, China). Invitrogen donated the TNF*α*-Mouse ELISA kit (#BMS607-3TEN). Each assay was carried out three times.

### Cell viability

The manufacturer’s recommendations were followed while utilizing the Cell Counting Kit-8 test kit (CCK-8, #HY-K0301, MedChemExpress) to determine the viability of the cells. To NRCMs, various dosages of Dox, shikonin, and Dox and shikonin were introduced. Using a spectrophotometer, the cell viability was determined at 570 nm (MULTISKAN. GO, Thermo Fisher Scientific, CA, USA). Cell viability was calculated using the formula shown below: (Absorbance of the experiment samples/Absorbance of the control) times 100 = percentage of cell viability. Untreated cells were thought to have a 100% viability rate.

### Western blotting and quantitative RT-PCR

Real-Time PCR from NRCMs or mouse hearts, protein samples were taken. Proteins were transferred to PVDF membranes after SDS-PAGE separation. The membranes were incubated with the appropriate primary antibodies overnight at 4 °C after being blocked with 5% non-fat milk at room temperature for 2 h. The bands were then cleaned and treated with secondary antibodies for 2 h at room temperature. Finally, the bands were observed using the ECL kit (Yeasen, Shanghai, China), and the protein levels were calculated using Image J. It was shown the relative protein expression adjusted to control. Antibodies used in the study: p67 phox (CST, #3923); SOD2 (ABCAM, ab68155); GAPDH (CST, # 5174S); p-p65 (ABCAM, ab194726); t-p65 (ABCAM, ab16502); Histone H3 (CST, # 9715); Bax (CST, # 9715); Bcl-2(ABCAM, ab196495); Nrf2 (ABCAM, ab137550); Ho1(ABCAM, ab13243); p-AMPK (CST, # 2535); t-AMPK (CST, #2603p); p-AKT (CST, # 4060); t-AKT (CST, # 4691);Mst1 (CST, # 3682).

Using a TRIzol reagent, total mRNA was isolated from cardiac tissues (Invitrogen, Carlsbad, USA). Using the Advantage® RT-for-PCR Kit (#639505, Takara Bio, Kusatsu, Shiga, Japan), the extracted RNA was reverse-transcribed into complementary DNA. SYBR Green Real-Time PCR Master Mix kit (#QPK-201, Takara, Dalian, China) was used for quantitative PCR utilizing an iQ5 Multi-Color Real-Time PCR Detection System (Bio-Rad, CA, USA).

### ROS assay

According to the manufacturer’s instructions, a fluorescent 2′,7′-dichlorofluorescindiacetate probe (H_2_DCF-DA, Thermo Fisher Scientific, Waltham, MA, USA) was used to measure the intracellular ROS levels. In brief, the H_2_DCF-DA given to the cells was incubated for an additional 30 min at 37 °C after the various treatments. Using a fluorescent microplate reader (Olympus BX51, Olympus, Optical Co. Ltd, Tokyo, Japan), the level of fluorescence was determined. Using ImageJ 1.47i software, the mean fluorescence intensity (MFI) from five random fields was calculated and utilized as a measure of ROS concentration.

### TUNEL assay

Sections of frozen heart tissue were divided, and they were then fixed in 4% neutral paraformaldehyde. The in situ apoptosis detection kit (Takara Bio Inc., Shiga, Japan) was used for the TUNEL assay, and these slides were examined under a fluorescence microscope. One individual who was unaware of the treatment group conducted this evaluation. In ice-cold PBS, heart tissues were homogenized.

### Statistical analysis

The software SPSS 20.0 was used to analyze all data, which was presented as mean ± standard error of mean (SEM). The unpaired Student’s t-test was used to establish the statistical comparison between the two groups. To compare differences between several groups, we utilized one-way analysis of variance followed by the Tukey test. At *p* < 0.05 or less (including *p* < 0.01 *p* < 0.001, etc.), differences were deemed statistically significant.

### Ethical approval

The Institutional Animal Care and Use Committee of Renmin Hospital of Wuhan University (Wuhan, China) authorized all animal experimentation techniques. The ethical committee at Renmin Hospital of Wuhan University accepted this study, and all experimental procedures were carried out in compliance with their institutional policies regarding the care and use of animals (Wuhan, China).

### Shikonin alleviated DOX-induced cardiotoxicity in mice

To explore the roles of shikonin in cardiac function, cardiac injury and survival of DOX-treated mice, mice were subjected to a low-dose DOX treatment with or without shikonin. DOX administration resulted in deterioration of cardiac function in mice, as verified by the decreased ejection fraction (EF), shortening fraction (FS) and pressure decay (dp/dt) in left ventricles, which were all increased in mice treated with shikonin (Fig. 1B–F). Previous studies indicated that DOX application significantly decreased the body weight in cancer patients^29^, but intriguingly, we found that shikonin attenuated DOX-induced body weight loss in mice (Fig. 1G), which raises the possibility for its clinical use. Meanwhile, we also found that DOX injection decreased the ratio of heart weight to tibia length (HW/TL), which were significantly alleviated by shikonin administration (Fig. 1H). We measured cTnT, CK-MB, and LDH levels to evaluate cardiac injury and function. These biomarkers are commonly used indicators of myocardial damage and provided valuable information on the protective effects of shikonin against doxorubicin-induced cardiotoxicity. And we observed that shikonin significantly reversed the increased levels cTnT, CK-MB and LDH in DOX-treated mice (Fig. 1I–K). Additionally, we observed that Shikonin treatment led to an increase in the survival rate of the mice and a significant increase in their body weight (Fig. S1A–C). More importantly, we found that administration of shikonin showed no hepatic toxicity in mice, as evaluated by the serum concentrations of liver enzymes (Fig. S1D,E). And shikonin did not affect heart rates (Fig. S1F). Altogether, these findings demonstrate that shikonin protects against DOX-induced myocardial damage and dysfunction.

### Shikonin treatment inhibited myocardial oxidative stress in DOX-treated mice

**Figure 2:**
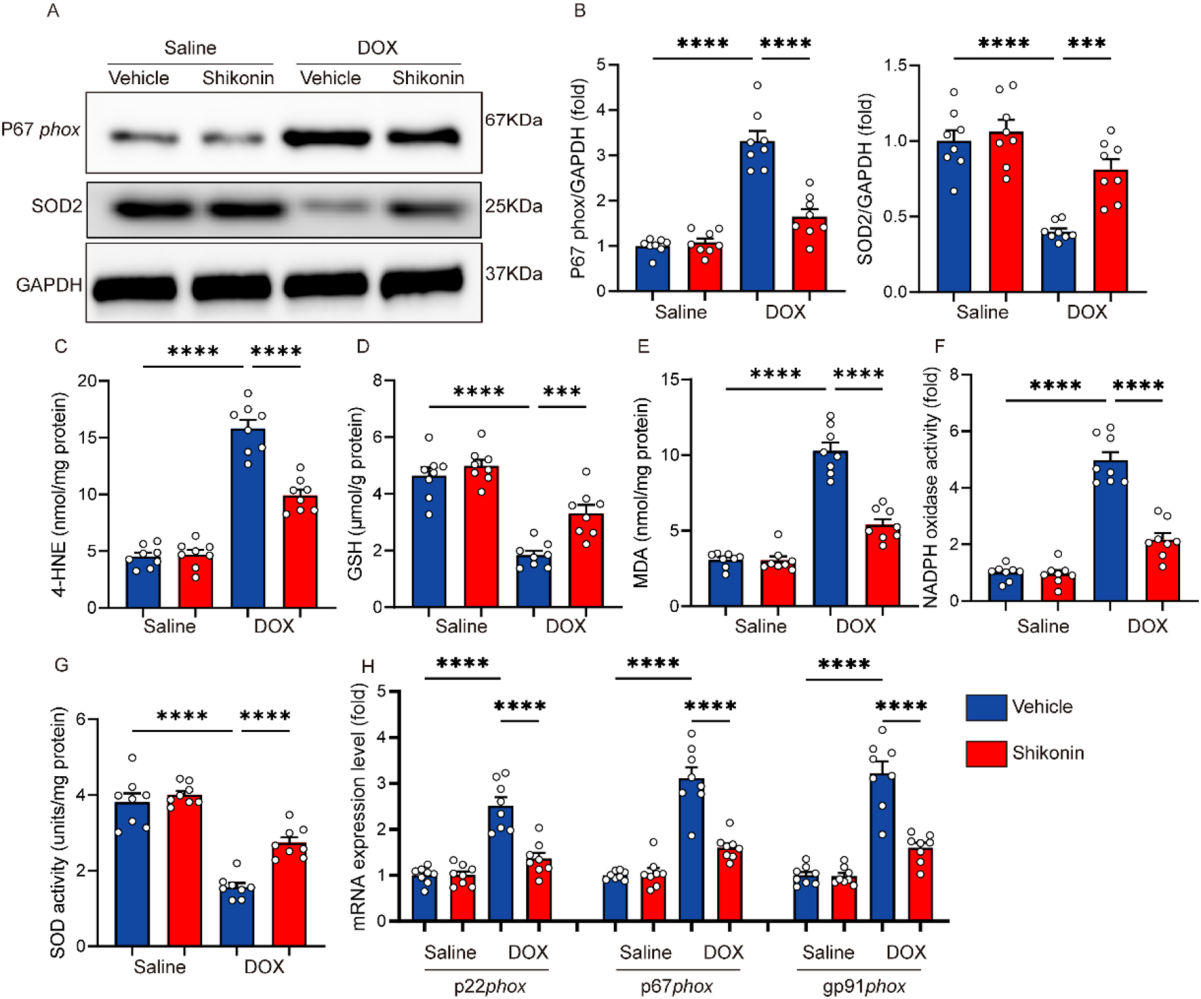
Shikonin attenuated DOX-induced oxidative stress in the hearts. (**A,B**) Western blot and quantitative analysis showing the protein levels of p67 phox and SOD2 in vehicle and shikonin treated mice (n = 8). Original blots/gels are presented in Supplementary Fig. S3. (**C–E**) Levels of 4-hydroxynonenal (4-HNE), endogenous antioxidants (GSH) content and malondialdehyde (MDA) in mice myocardium (n = 8). (**F**) NADPH oxidase activity (n = 8). (**G**) Total SOD activity in the myocardium (n = 8). H, NADPH oxidase subunits mRNA expression by real time RT-PCR (n = 8). ***p < 0.001, ****p < 0.0001, significantly different as indicated.

Oxidative stress was involved in the development of DOX-induced cardiotoxicity. Western blots showed that shikonin significantly reversed the up-regulation of NADPH oxidase subunit p67phox and down-regulation of SOD2 expression induced by dox treatment (Fig. 2A,B). In our study, we assessed several key oxidative stress indicators, including MDA level, 4-HNE level, GSH level, and total SOD activity, to comprehensively evaluate the oxidative status. Additionally, we measured NADPH oxidase activity as a crucial indicator of oxidative stress pathway activation. Consistent with the molecular changes, we observed that shikonin decreased the excessive MDA level, 4-HNE and NADPH oxidase activity caused by DOX, and increased the low GSH level and total SOD activity caused by DOX (Fig. 2C–G). In addition, the mRNA level of NADPH oxidase subunit (p67phox, p22phox and gp91phox) decreased significantly after shikonin administration (Fig. 2H).

### Shikonin modulated DOX-induced inflammation and apoptosis in vivo

**Figure 3:**
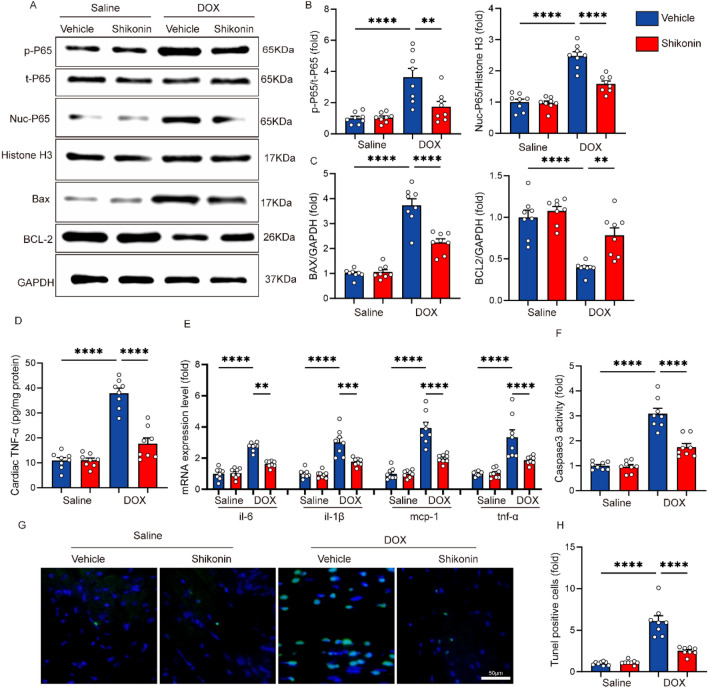
Shikonin attenuated DOX-induced cardiomyocyte inflammation and apoptosis. (**A–C**) Western blot and quantitative analysis showing the protein levels of p-P65, t-P65, Nuc-P65, Bax, Bcl-2 in four groups (n = 6). Original blots/gels are presented in Supplementary Fig. S4. (**D**) Cardiac TNF-α levels as detected by ELISA (n = 8). (**E**) The relative mRNA levels of il-6, il-1β, tnf-α, and mcp-1 normalized to gapdh in mice (n = 8). (**F**) Activity of caspase-3 of mice in four groups (n = 8). (**G,H**) Myocardial apoptosis measured by TUNEL staining in heart sections (n = 8, bar = 50 μm). **p < 0.01, ****p < 0.0001, significantly different as indicated.

Next, we detected proteins that are representative of inflammation and apoptosis. In our research, we examined the levels of p65 and TNF-α to understand the inflammatory response in the context of doxorubicin-induced cardiotoxicity. These indicators served as crucial markers in elucidating the inflammatory pathways involved in our experimental model. Western bolt analysis indicated that DOX promoted the phosphorylation and nuclear accumulation of p65, and shikonin administration mostly reversed this alteration (Fig. 3A,B). Cardiac TNF-α levels detected by ELISA showed that shikonin decreased the elevlated cardiac TNF-α induced by DOX (Fig. 3D). The mRNA expression of cardiac inflammation biomarkers were also examined, and the result showed that DOX administration promoted the mRNA expression of pro-inflammatory genes (il-6, il-1β, mcp-1 and tnf-α), which were prevented by shikonin treatment (Fig. 3E). In our study, we investigated the expression of key apoptotic regulators, including Bax and Bcl-2, along with assessing caspase-3 activity, to elucidate the underlying mechanisms of cell apoptosis in response to doxorubicin-induced cardiotoxicity. Shikonin attenuated DOX-induced upregulation of Bax and the down-regulation of Bcl-2 (Fig. 3A,C). Besides, shikonin significantly reduced the level of apoptotic cardiomyocytes in DOX-treated mice, as evidenced by Tunel staining and caspase3 activity (Fig. 3F–H).

### Nrf2 knockdown counteracted the inhibitory effects of shikonin on oxidative stress, apoptosis and inflammatory in vitro

**Figure 4:**
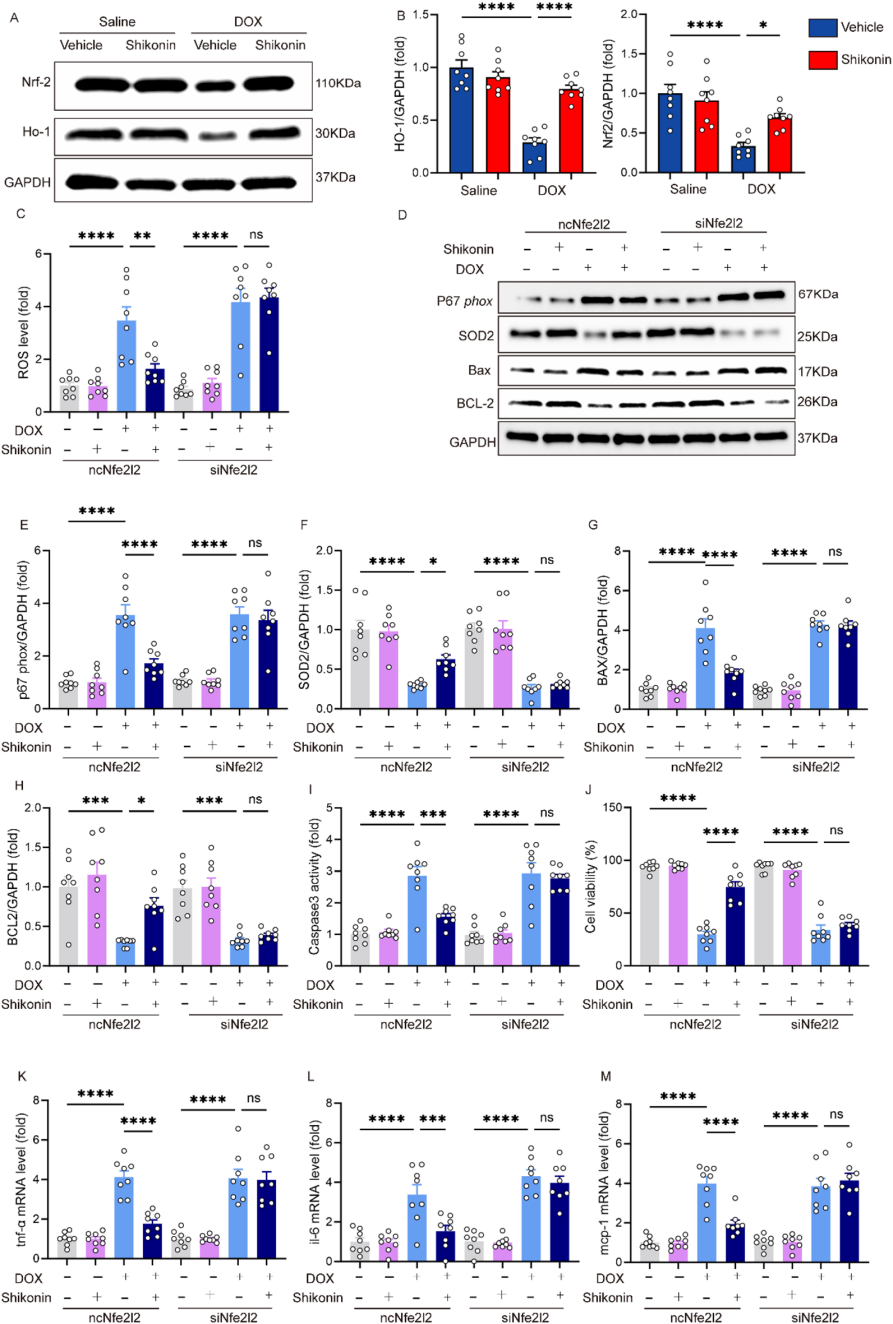
Nrf2 knockdown counteracted the inhibitory effects of shikonin on oxidative stress, apoptosis and inflammatory in vitro. (**A,B**) Western blot and quantitative analysis showing the protein levels of Nrf2 and HO-1 among different groups (n = 6). Original blots/gels are presented in Supplementary Fig. S5. (**C**) ROS level (n = 8). (**D–H**) Western blot and quantitative analysis showing the protein levels of p67 phox, SOD2, Bax, Bcl-2 in vitro (n = 6). Original blots/gels are presented in Supplementary Fig. S6. (**I**) Activity of caspase-3(n = 6). (**J**) CCK-8 assay for cell viability (n = 6). (**K–M**) The relative mRNA levels of tnf-α, il-6 and mcp-1 normalized to gapdh in vitro (n = 6). *p < 0.05, **p < 0.01, ***p < 0.001, ****p < 0.0001, significantly different as indicated. *ns* not significant.

Next, we investigated the precise mechanisms by which shikonin protected against DOX-induced cardiotoxicity. We investigated the Nrf2 expression and its downstream target, HO-1, to assess the activation of the antioxidant response pathway and its role in mitigating doxorubicin-induced cardiotoxicity. These indicators provided insights into the cellular mechanisms underlying the protective effects of shikonin. Compared with the control group, mice treated with DOX exhibited decreased expression of Nrf2 and HO-1 in the hearts, and shikonin significantly increased the expression of Nrf2 and HO-1 (Fig. 4A,B). Given the results that shikonin activated Nrf2 in vitro, we further assessed whether Nrf2 was involved in the protective effects of shikonin on DOX-induced cardiotoxicity. NRCMs were transfected with siRNA to silence Nrf2 (Fig. S2A). Surprisingly, Nrf2 knockdown blocked the protection afforded by shikonin against ROS production. ROS level and the expression of p67phox and SOD2 confirmed that shikonin alleviated DOX-induced ROS by enhancing expression of Nrf2 (Fig. 4C–F). Next, we assessed whether shikonin protected against DOX-induced death of myocytes. shikonin attenuated DOX-induced upregulation of Bax and the down-regulation of Bcl-2 in NRCMs (Fig. 4D,G,H); Shikonin administration increased cell viability and decreased caspase3 activity NRCMs treated with DOX, however, these changes were blocked by shikonin treatment. The effects of shikonin were abolished by Nrf2 knockdown (Fig. 4I–J). Given the inhibitory role of shikonin on inflammation in vivo, we then detected whether shikonin could affect DOX-induced inflammation in vitro. NRCMs treated with DOX and shikonin had lower mRNA levels of il-6, mcp-1 and tnf-α when compared with DOX-treated cells. However, these changes in these levels were blunted by Nrf2 knockdown (Fig. 4K–M).

### Nrf2 knockdown counteracted the protective effects of shikonin on cardiac injury and dysfunction caused by DOX in mice

**Figure 5:**
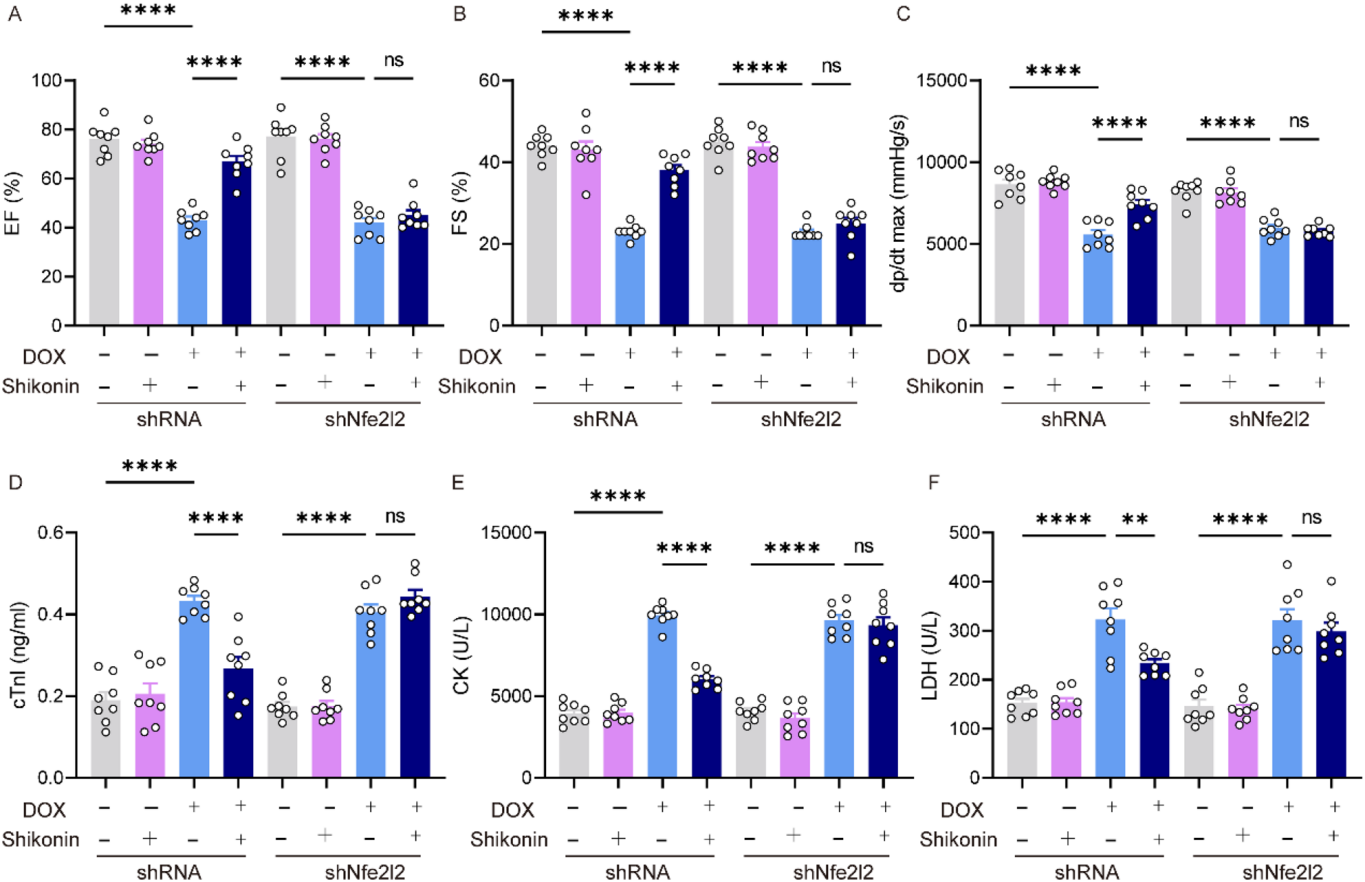
Nrf2 knockdown blocked the protection of shikonin against DOX-induced cardiac injury in vivo. (**A–C**) Left ventricular ejection fraction (EF%), left ventricular fraction shortening (FS%) and maximal left ventricular pressure rising rate (dp/dt max) were quantified via echocardiography (n = 8). (**D–F**) Plasma cTnI (cardiac troponin I), creatine kinase (CK) and lactated hydrogenase (LDH) concentrations in mice were measured by ELISA (n = 8). *p < 0.01, ****p < 0.0001, significantly different as indicated. *ns* not significant.

**Figure 6:**
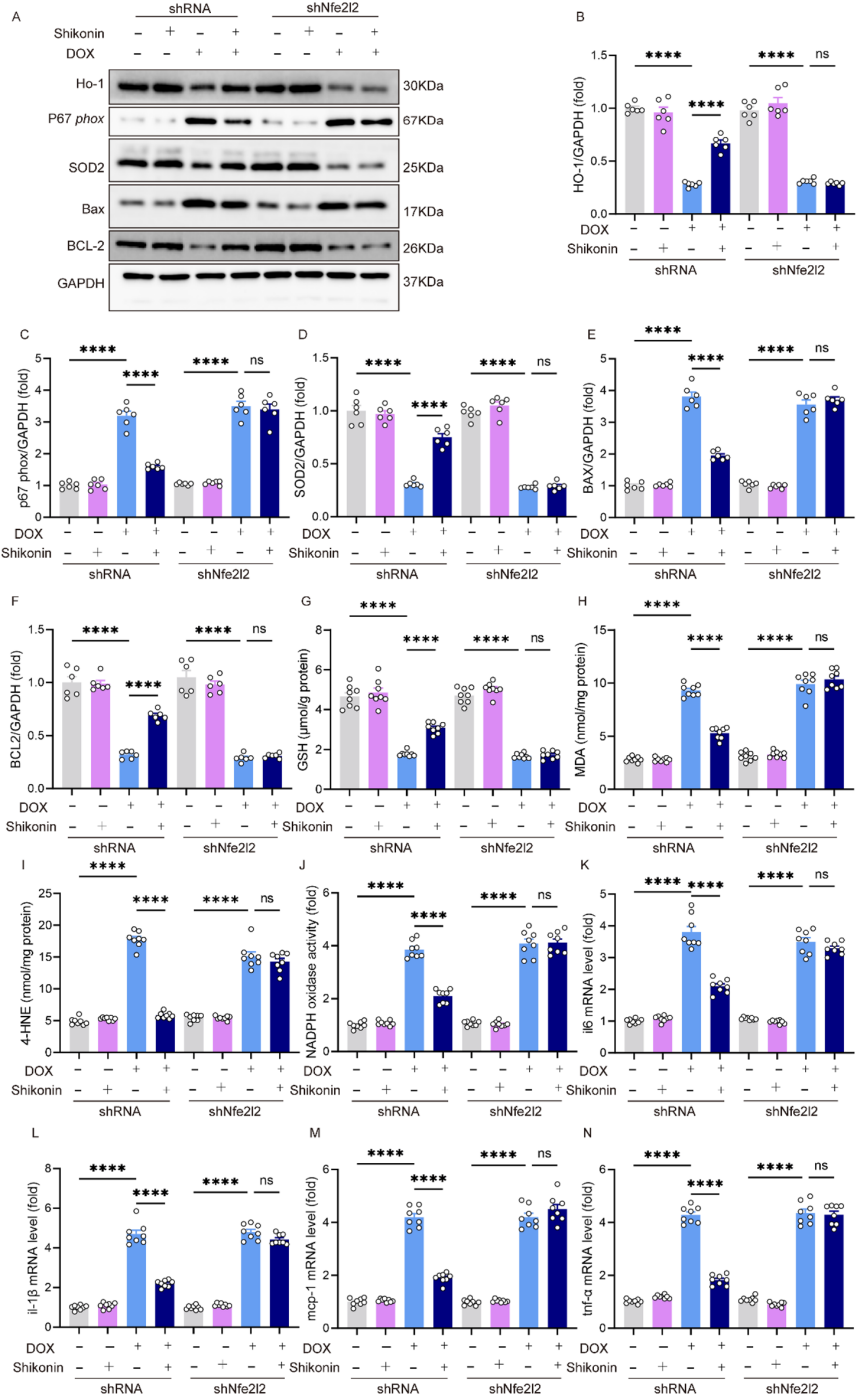
Nrf2 knockdown blocked the protection of shikonin against DOX-induced oxidative stress, apoptosis and inflammation in hearts. (**A–F**) Western blot and quantitative analysis showing the protein levels of HO-1, p67 phox, SOD2, Bax, Bcl-2 in viVo (n = 6). Original blots/gels are presented in Supplementary Fig. S7. (**G–J**) Levels of endogenous antioxidants (GSH), malondialdehyde (MDA), 4-hydroxynonenal (4-HNE) and NADPH oxidase activity in mice myocardium (n = 8). (**K–N**) The relative mRNA levels of il-6, il-1β, mcp-1and tnf-α, il-6 normalized to gapdh in vivo (n = 6). **p < 0.01, ****p < 0.0001, significantly different as indicated. *ns* not significant.

To confirm the role of Nrf2 in shikonin’s protective effects on cardiac injury and dysfunction, we used Adv to deliver shNfe2l2 to knock down Nrf2 expression in mice (Fig. S2B). Shikonin lost its protective effects against DOX-induced cardiac dysfunction and cardiac injury, as evidenced by unaltered FS, ± dp/dt, cTnI, CK-MB and LDH (Fig. 5A–F). The oxidative effect was evaluated in Nrf2 knockdown mice in vivo. Excessive oxidative stress was observed in DOX-treated mice hearts as evidenced by decreased HO-1, SOD2 and GSH and increased MDA, 4-HNE and NADPH oxidase activity. However, upon Nrf2 knockdown, these alterations were not significantly different between vehicle-treated and shikonin-treated mice hearts (Fig. 6A–D,G–J). In addition, shikonin lost its protection against cell apoptosis in Nrf2 knockdown mice as evidenced by unchanged expression of Bcl-2 and Bax (Fig. 6A,E,F). The expression levels of il-6, il-1β, mcp-1 and tnf-α mRNA demonstrated Nrf2 knockdown abrogated the shikonin-mediated protection against myocardial inflammation (Fig. 6K–N).

### The mechanism by which shikonin activated Nrf2

**Figure 7:**
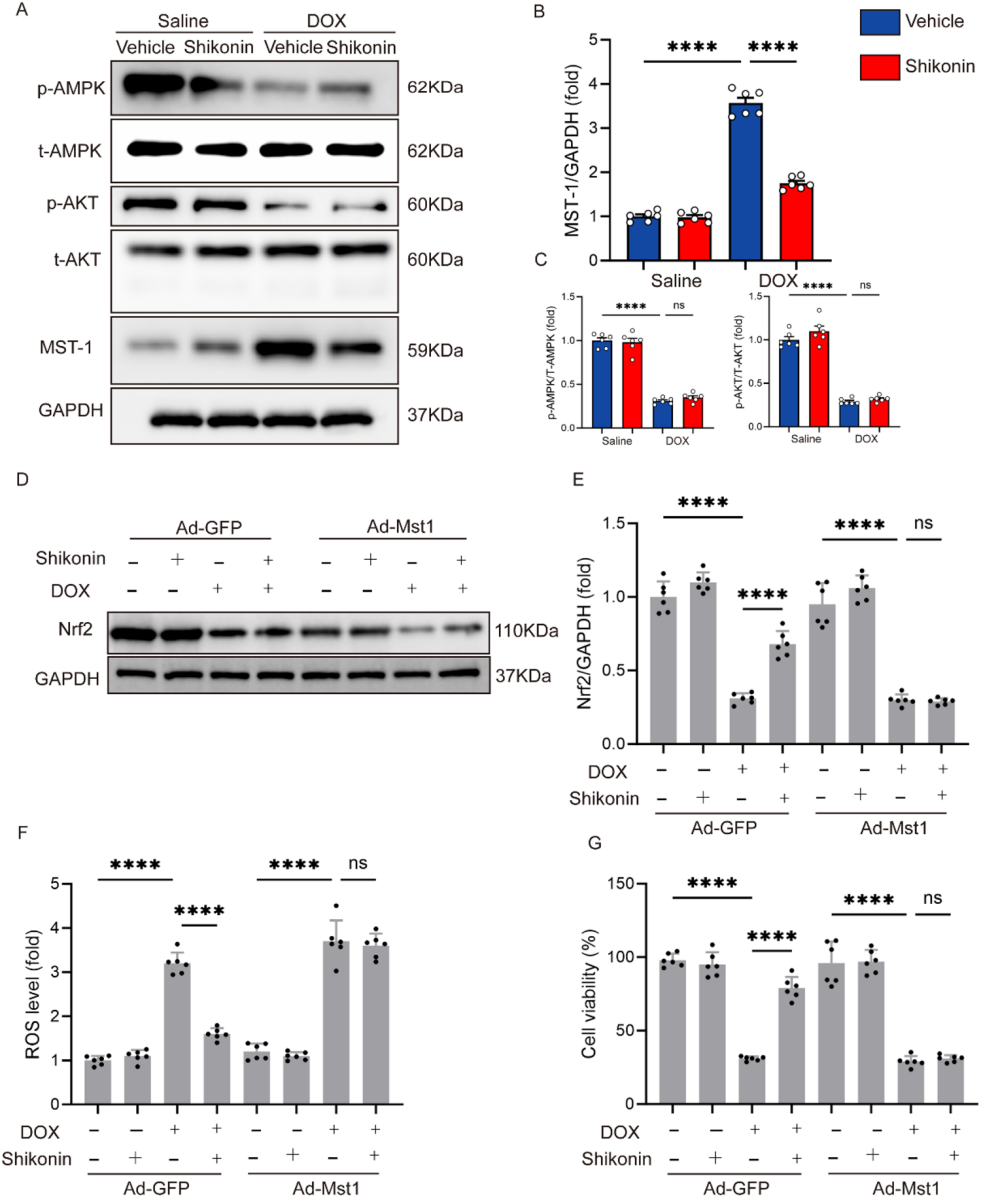
Mst1 downregulation was responsible for shikonin-mediated activation on Nrf2. (**A–C**) Western blot and quantitative analysis showing the protein levels of p-AMPK, t-AMPK, p-AKT, t-AKT and Mst1 in vivo (n = 6). Original blots/gels are presented in Supplementary Fig. S8. (**D,E**) Western blot and quantitative analysis showing the Nrf2 expression after Ad-Mst1administration (n = 6). Original blots/gels are presented in Supplementary Fig. S9. (**F**) ROS level (n = 6). (**G**) CCK-8 assay for cell viability (n = 6). **p < 0.01, ****p < 0.0001, significantly different as indicated. *ns* not significant.

Next, we explored the precise mechanism by which shikonin activated Nrf2. One of the essential elements of the Hippo signaling system, which regulates cell survival and death to control tissue growth, is Mst1^30^. Mst1 is well known for being a pro-apoptotic molecule, and by being suppressed, it reduces the generation of ROS, which lessens cell apoptosis^31^. Recent research has revealed that Mst1 controls both autophagy and apoptosis in cardiomyocytes^32,33^. We detected the expression of Mst1 and found that the high expression of Mst1 induced by DOX was significantly decreased after shikonin administration (Fig. 7A,B). Compared with the control groups, mice treated with DOX displayed decreased phosphorylation of AMPK and AKT in the hearts, but shikonin couldn’t increase the phosphorylation of AMPK and AKT (Fig. 7A,C). To Verify the hypothesis that shikonin activated Nrf2 via Mst1, NRCMs were infected with adenovirus to overexpress Mst1 (Fig. S2C). In the cells infected with GFP, shikonin could reverse the low expression of Nrf2 induced by DOX, but in the cells overexpressing Mst1, shikonin had no effect on the low expression of Nrf2 induced by DOX (Fig. 7D,E). Further detection of ROS level showed that shikonin decreased the level of ROS induced by DOX, and overexpression of Mst1 completely offsets the protective effect of shikonin on cardiomyocyte oxidative stress (Fig. 7F). In addition, NRCMs exposed to DOX had decreased cell viability and after shikonin administration the cell viability was increased. However, overexpression of Mst1 abolished the protection of shikonin against DOX-induced cell death (Fig. 7G).

## Discussion

The present study demonstrates that shikonin has a significant protective effect on doxorubicin-induced cardiotoxicity both in vitro and in vivo by reducing inflammation, oxidative stress, and cardiomyocyte apoptosis. These effects were mediated by down-regulating Mst1 and subsequently upregulating Nrf2 expression. These findings provide important insights into the potential therapeutic application of shikonin in mitigating doxorubicin-induced cardiotoxicity.

DOX-induced cardiotoxicity is largely attributed to oxidative stress, resulting from the overproduction of electrophilic reagents and oxidants following DOX therapy. This oxidative stress can cause damage to the heart by oxidizing membrane lipids and generating highly reactive 4-HNE^34^. Previous studies have shown that shikonin possesses direct and indirect antioxidant properties, as evidenced by its ability to restore SOD expression and GSH levels, as well as block oxidative stress^35^. Additionally, Zhang et al. demonstrated that shikonin significantly attenuates the oxidative stress caused by angiotensin II during cardiac remodeling^24^. In vivo studies have shown that shikonin can reduce ROS levels and mitigate DOX-induced oxidative stress by increasing the expression of SOD and p67phox, as well as the quantities of GSH, MDA, and 4-HNE in cardiac tissue. This protective response is attributed to shikonin’s inherent radical-scavenging ability.

The cardiotoxicity induced by doxorubicin has been associated with various types of regulated apoptosis and inflammation. Pretreatment with shikonin has been shown to significantly reduce Bax expression, increase Bcl-2 expression and the Bcl-2/Bax ratio, and markedly suppress apoptosis^25,36^. Shikonin has also been found to significantly decrease the production of TNF-α, IL-6, and IL-1 induced by acetaminophen, and to suppress the expression of genes involved in inflammation^35^. Consistent with these findings, we observed a marked increase in myocardial apoptosis and inflammation following DOX administration. However, shikonin treatment significantly reduced the expression of inflammatory markers (phosphorylation and nuclear accumulation of p65, cardiac TNF-α, and so on), caspase activity, TUNEL-positive cells, and the Bax/Bcl-2 ratio in mice given DOX injections. These results indicate that shikonin therapy may prevent the development of inflammatory reactions and apoptotic changes induced by DOX.

Natural products have the potential to modulate the signaling pathways implicated in DOX-induced cardiotoxicity, in addition to their direct effects on the heart. Resveratrol, for instance, has been found to activate the Nrf2 signaling pathway, whereas curcumin and quercetin have been shown to inhibit the Mst1 signaling pathway^37,38^. Nrf2 deficiency has been found to exacerbate DOX-induced cardiac dysfunction and cardiotoxicity in mice, whereas pharmacological activation of Nrf2 has been found to protect against DOX toxicity by increasing the expression of antioxidant and antielectrophile enzymes^39,40^. Shikonin, as demonstrated by Hu et al., can enhance the expression of Nrf2 and its downstream targets, boosting the antioxidant capacity of spiral ganglion neuron and Schwann cells, as well as inhibiting cell apoptosis by activating the Nrf2/antioxidant response element (ARE) signaling pathway^41^. In our investigation, we observed that DOX exposure led to a reduction in the expression of Nrf2 and its downstream targets. Shikonin therapy, however, effectively reversed these pathological changes, indicating its potential in increasing antioxidant capacity in DOX-related heart damage via Nrf2 activation.

According to various reports, Mst1 has been linked to numerous types of heart injuries. Yamamoto et al. discovered that overexpression of Mst1 in the heart led to dilated cardiomyopathy in mice and that Mst1 was cleaved in response to proapoptotic stimulation in cardiomyocytes, contributing to cardiac dysfunction^42^. Mst1 inhibition has been shown to reduce cardiomyocyte apoptosis and improve cardiac function in response to myocardial infarction. This protein kinase is activated via C-terminal cleavage as a stress sensor in response to oxidative stress and apoptotic stimuli, ultimately leading to cell apoptosis^43^. Rab10 overexpression in the heart can reduce heart dysfunction and damage caused by DOX by preventing Mst1 activity^44^. Here, we demonstrate for the first time that shikonin therapy can deactivate Mst1 and offer protection against DOX-induced cardiac damage. Mst1 gene loss can reduce the self-protective capability of the Keap1/Nrf2 axis against oxidative stress in macrophages^45^. According to Zhang et al., Mst1 inhibition in bone marrow mesenchymal stem cells has protective effects because it activates the Keap1/Nrf2 signaling pathway^46^. A recent study elucidated that Mst1 overexpression leads to Nrf2 downregulation, forming the Nrf2-Keap1 complex^47^. In our investigation exploring the engagement of Mst1 within the Nrf2 pathway during anti-oxidative processes in DOX-injured cells, antioxidant process of dox cardiotoxicity, and adenoviral overexpression of Mst1 revealed alterations in Nrf2 under DOX stress conditions. Overexpression of Mst1 inhibited Nrf2 expression. This indicated a potential positive regulatory role of Mst1 in the Nrf2 pathway. However, contradictory findings in other studies demonstrated that the significance of the Mst1-Nrf2 axis in ROS detection and antioxidant mechanisms in macrophages^45^. This research elucidated that ROS activate Mst1/2 to phosphorylate Keap1, impeding its aggregation, thereby obstructing Nrf2 ubiquitination and degradation. Discrepancies in this mechanism of Mst1-Nrf2 could stem from several reasons. Firstly, differences in the cell types studied might contribute to these disparities. Another possibility is that the overexpression of Mst1 might not affect its phosphorylation activity, thus failing to impede Nrf2 ubiquitination and degradation. Additional experiments to assess Mst1 phosphorylation activity might be necessary to elucidate this aspect. Another study may be more illustrative of the complex mechanism between Mst1-Nrf2. This study found that Nrf2 expression increased after Mst1 depletion, whereas Nrf2 silencing abolished the protective effect of Mst1 depletion on nasal epithelial survival and mitochondrial homeostasis^48^. In addition, Mst1 overexpression effectively reversed shikonin-induced Nrf2 upregulation while leading to increased ROS production. Studies have consistently shown that reduced Nrf2 activity leads to reduced cell survival in cardiomyocytes under hypoxic conditions^49^. This suggests that Mst1 overexpression may inactivate the Nrf2 pathway and aggravate the severe cellular damage caused by oxidative stress.

## Conclusions

Based on the findings of this study, we propose that shikonin may be a promising natural product for mitigating doxorubicin-induced cardiotoxicity. Shikonin could be used as an adjuvant therapy to enhance the efficacy and reduce the side effects of doxorubicin chemotherapy. Alternatively, shikonin could be used as a preventive agent to protect healthy individuals from environmental or occupational exposure to doxorubicin or other cardiotoxic agents. However, before clinical application, more preclinical studies are required to confirm its pharmacological properties and safety profile.

### Supplementary Information

Supplementary Figures.

## Data Availability

The data that support the findings of this study are available from the corresponding author upon reasonable request.

## Abbreviations

AdV: Adenoviral vectors
DOX: Doxorubicin
EF: Ejection fraction
GSH: Glutathione
Mst1: Mammalian sterile 20-like kinase 1
MDA: Malondialdehyde
Nrf2: Nuclear factor erythroid 2-related factor 2
NRCMs: Neonatal rat cardiomyocytes
SOD: Superoxide dismutase
TNF-*α*: Tumor necrosis factor-α
FS: Shortening fraction
