# Supplementary material for: Shikonin alleviates doxorubicin-induced cardiotoxicity via Mst1/Nrf2 pathway in mice
Source: Sci Rep. 2024 Jan 9;14:924. doi: 10.1038/s41598-024-51675-7 (PMC10776756; doi:10.1038/s41598-024-51675-7)
Supplement: Supplementary file 1 — Supplementary Figures. [file 41598_2024_51675_MOESM1_ESM.docx]

**Shikonin Alleviates** **Doxorubicin-Induced Cardiotoxicity via Mst1/Nrf2 Pathway in mice**

Hu Tuo^1*^, Wenjing Li^1^, Wei Zhao^1^, Juan Zhao^1^, Danni Li^2^, Lin Jin^3#^

^1^Department of Pediatrics, Renmin Hospital of Wuhan University, Wuhan China

^2^Department of Anesthesiology, Renmin Hospital of Wuhan University, Wuhan China

^3^Department of Orthopedics, Renmin Hospital of Wuhan University, Wuhan China

Corresponding author:

Lin Jin

Department of Orthopedics, Renmin Hospital of Wuhan University,

Wuhan University at Jiefang Road 238, Wuhan 430060, RP China

E-mail: jinlin2010@whu.edu.cn


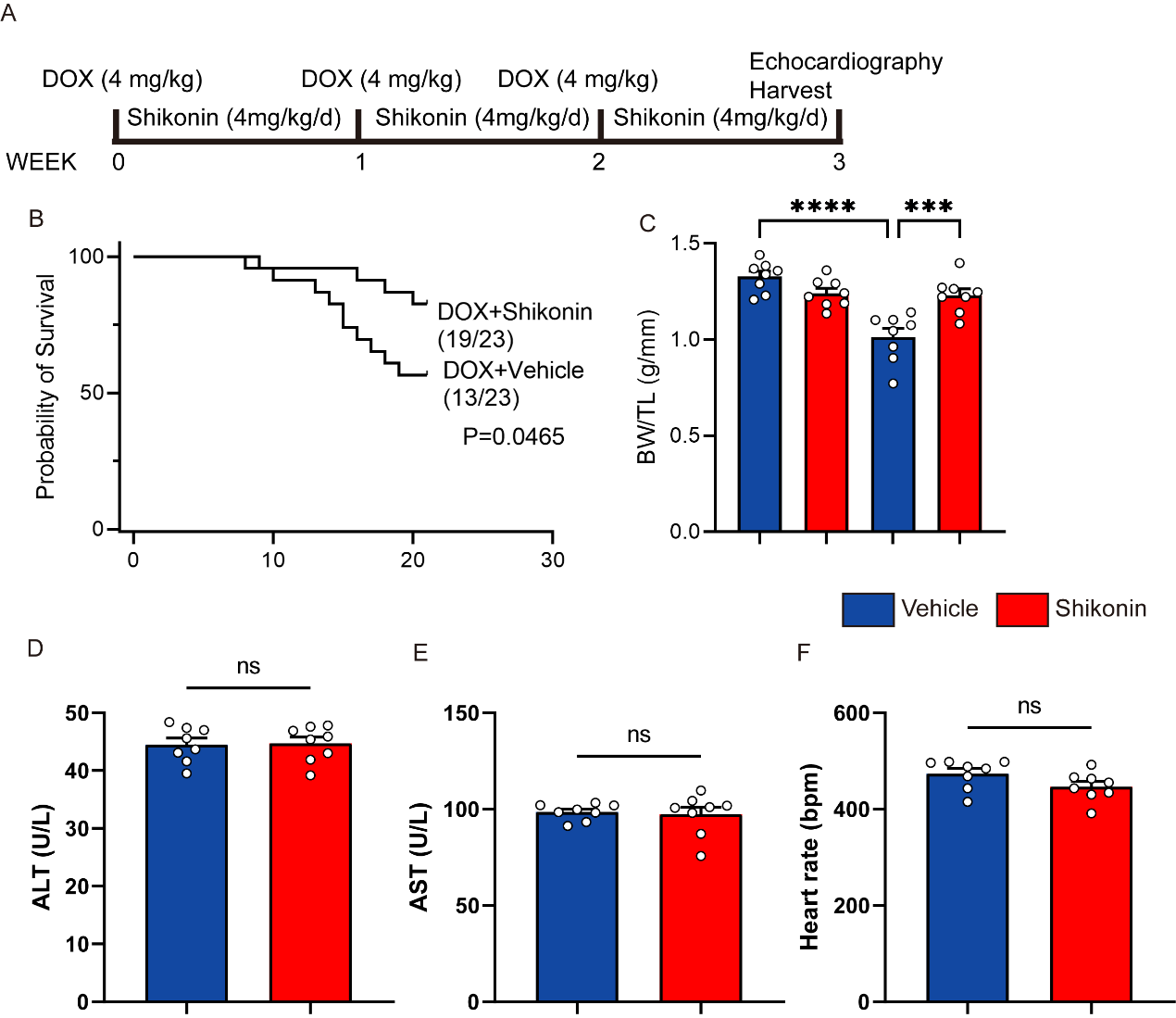


Figure S1. Shikonin improved body weight and survival rate after DOX treatment in mice, but do not affect liver function, heart rate.

A. The treatment schedule. B. Kaplan-Meier survival analysis showing improved survival in shikonin treatment compared with Veh after DOX treatment. C. Body weight normalized to the tibial length (n=8). D-E, Plasma glutamic pyruvic transaminase (ALT) and glutamic oxaloacetic transaminase (AST）concentrations in mice were measured by ELISA (n=8). F, Heart rates in mice (n=8). ****P*< 0.001, *****P*< 0.0001, ns, Not significant.


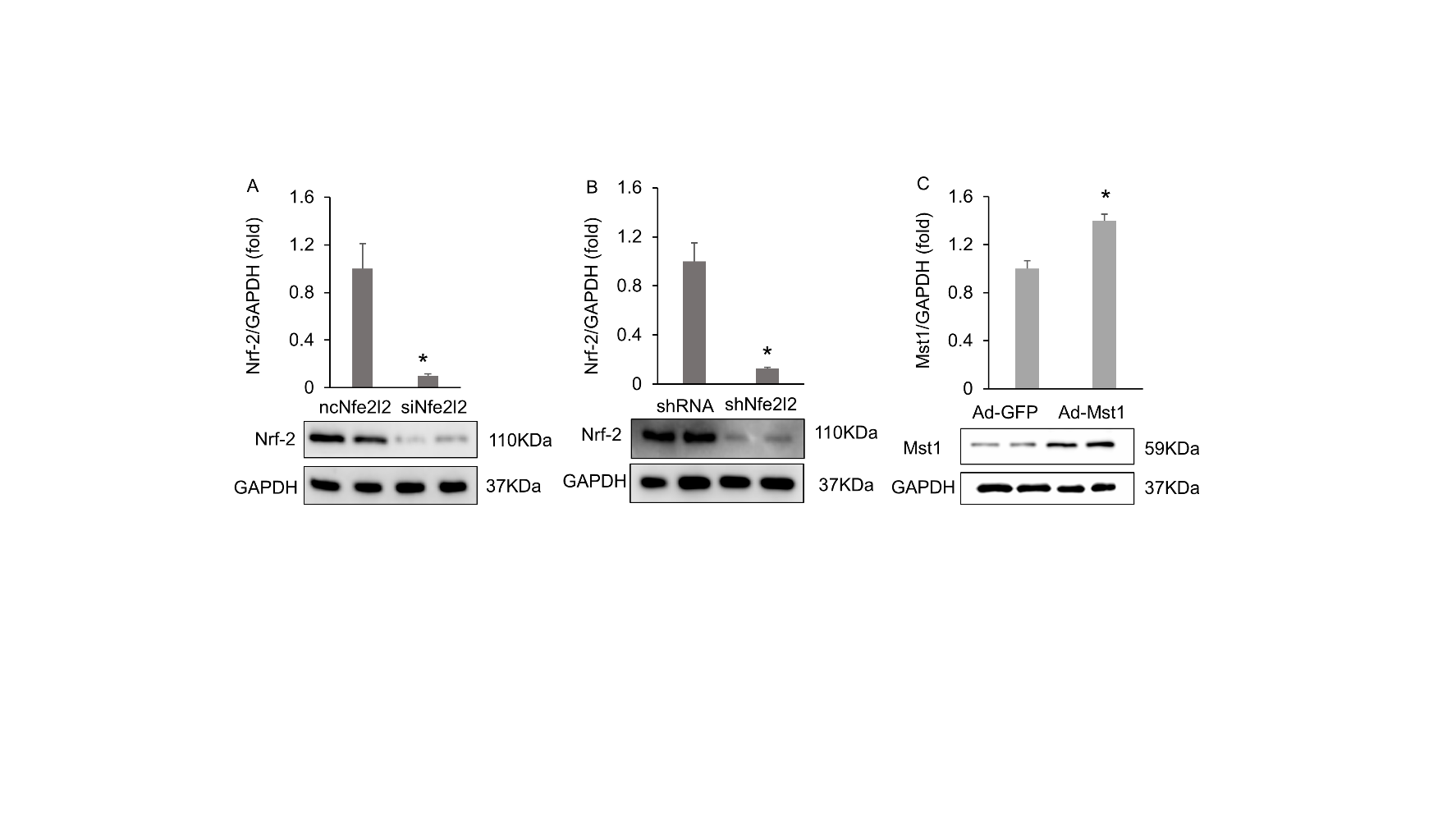


Figure S2. Validation of Nrf-2 and Mst1 expression was performed by Western blot. A. siNfe2I2 was transfected into NRCMs, and then Nrf2 expression was detected and quantified by Western blot (n=3). Original blots/gels are presented in Supplementary Figure S10. B. Adenoviral vectors were used to target the heart to deliver shNfe2l2, and Nrf2 expression was detected and quantified by Western blot (n=3). Original blots/gels are presented in Supplementary Figure S11. C. NRCMs was infected with adenovirus to overexpress Mst1, and the expression of Mst1 was detected and quantified by Western blot (n=3). Original blots/gels are presented in Supplementary Figure S12. ****P*< 0.05.

Origin gels


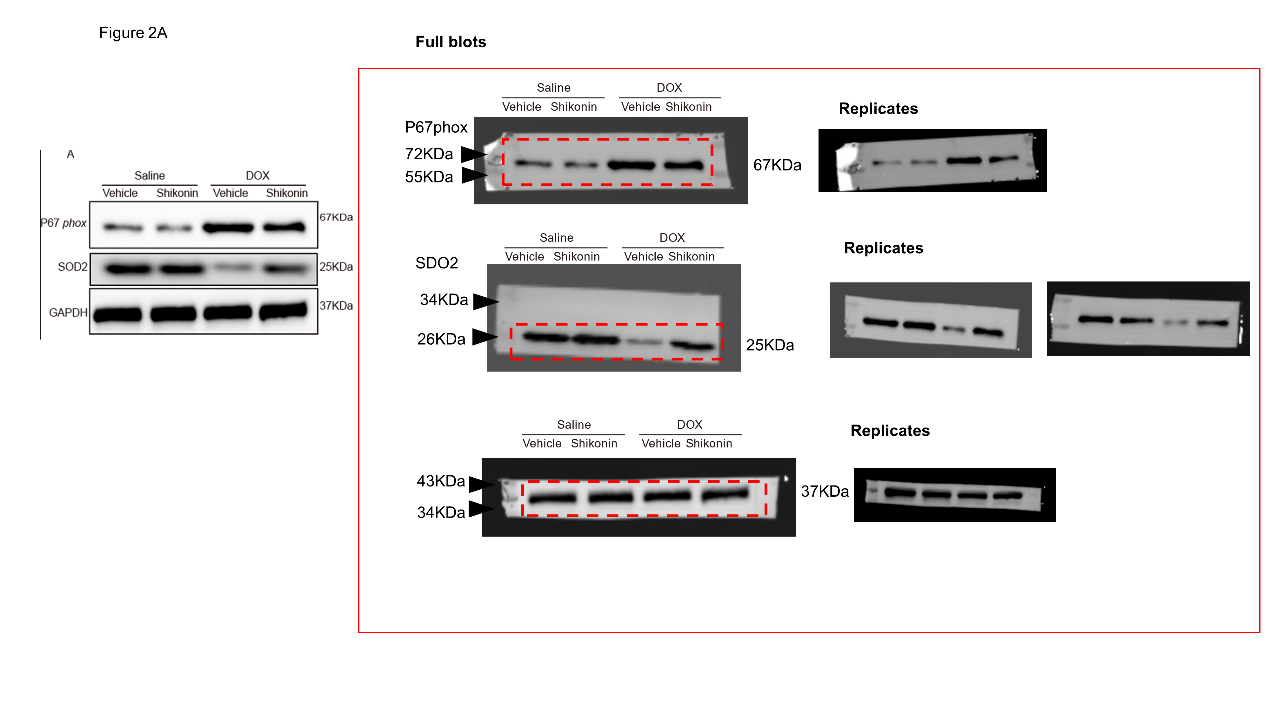


Figure S3. Original blots/gels of Figure2A.


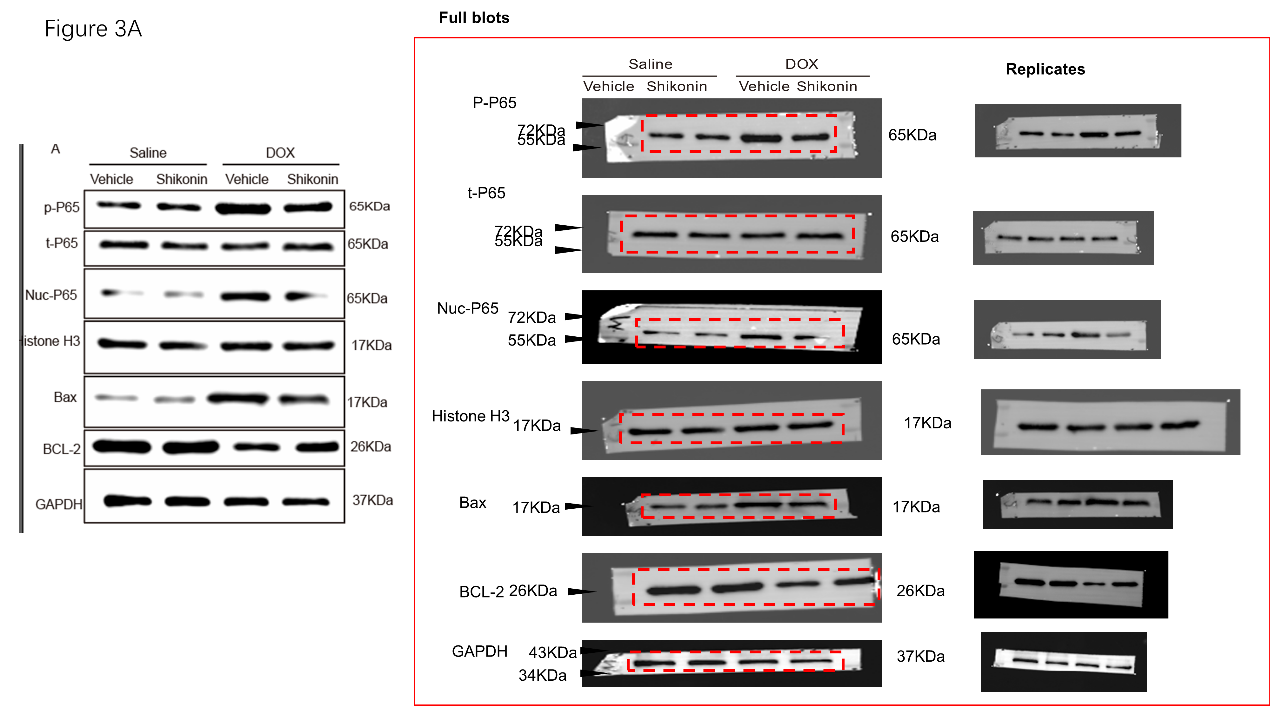


Figure S4. Original blots/gels of Figure3A.


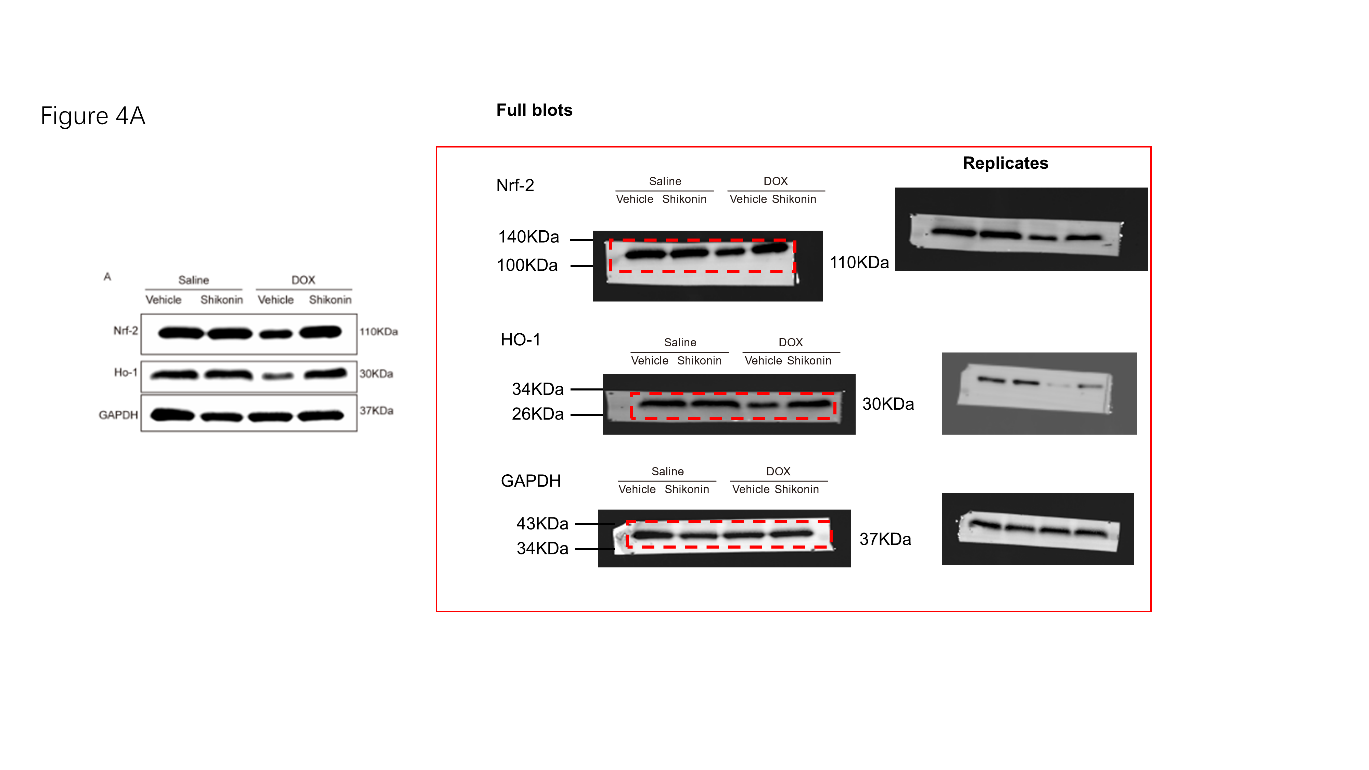


Figure S5. Original blots/gels of Figure4A.


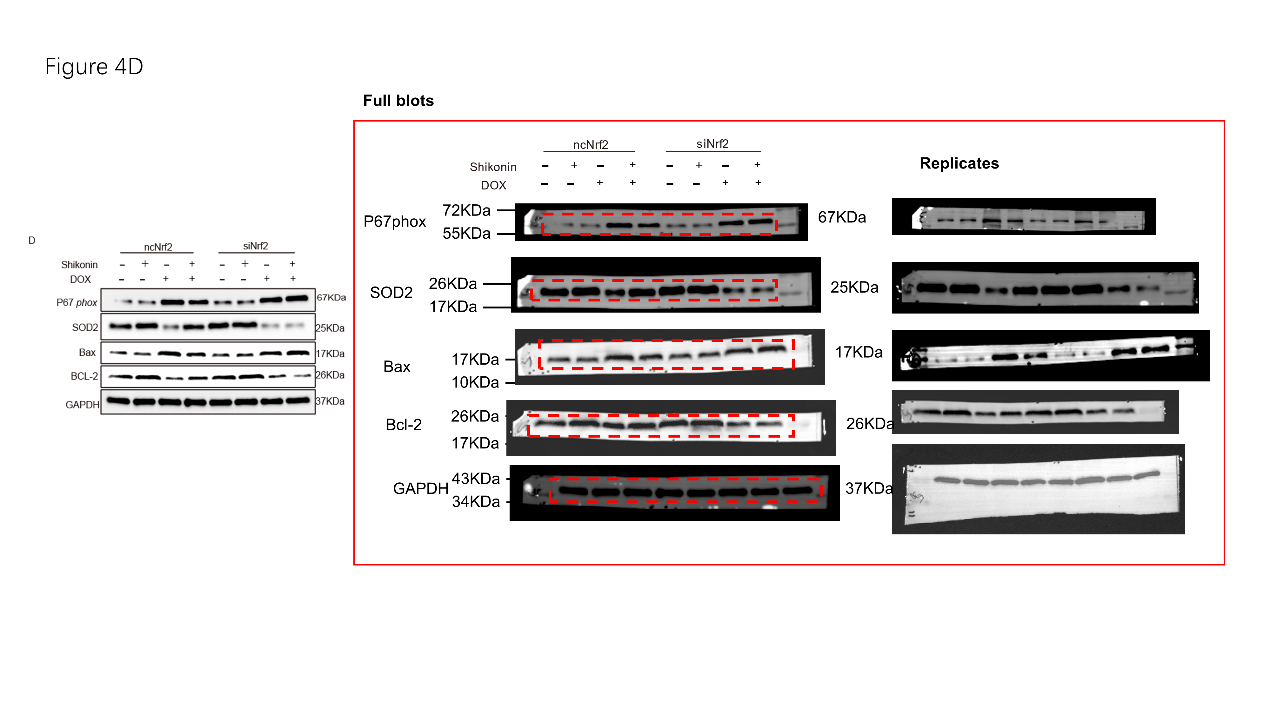


Figure S6. Original blots/gels of Figure4D.


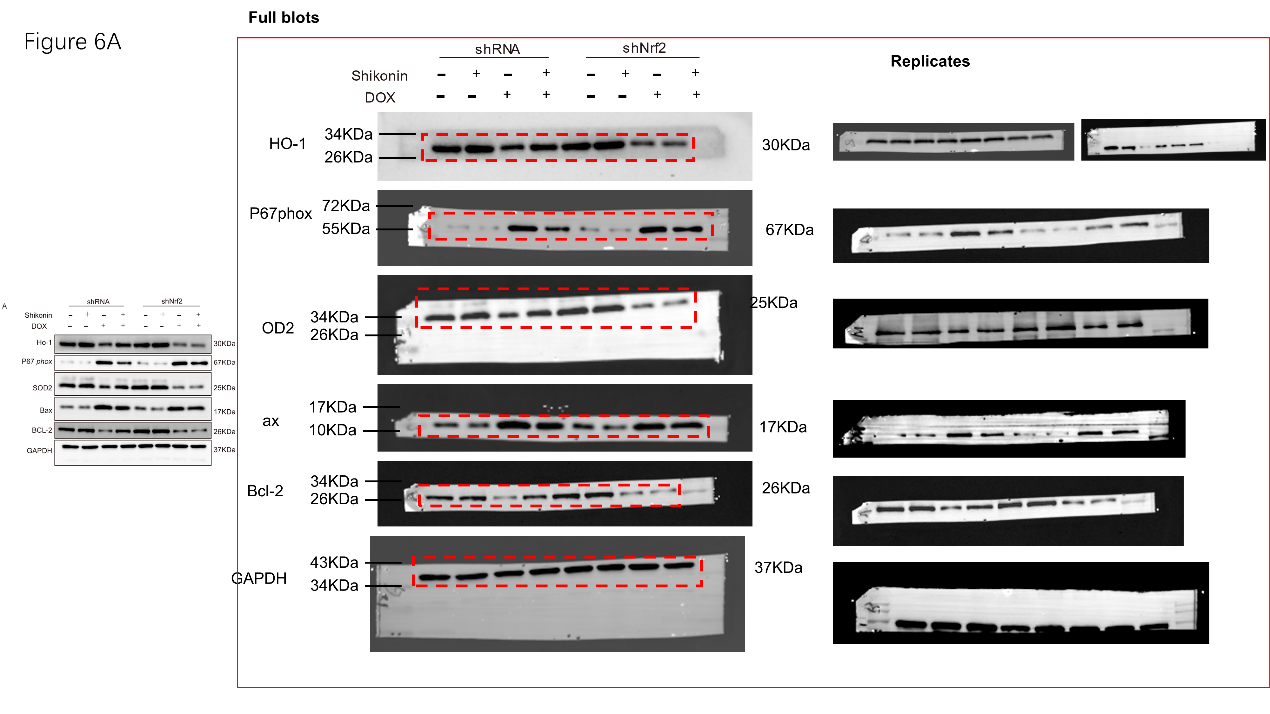


Figure S7. Original blots/gels of Figure6A.


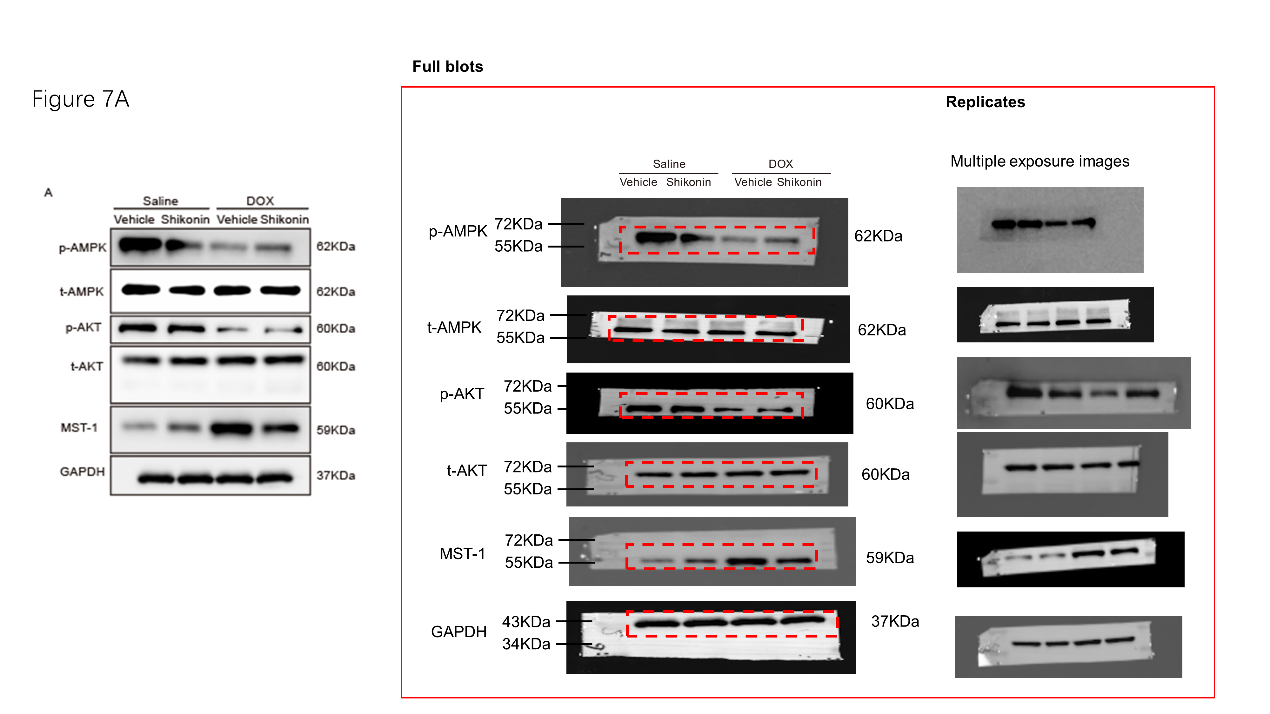


Figure S8. Original blots/gels of Figure7A.


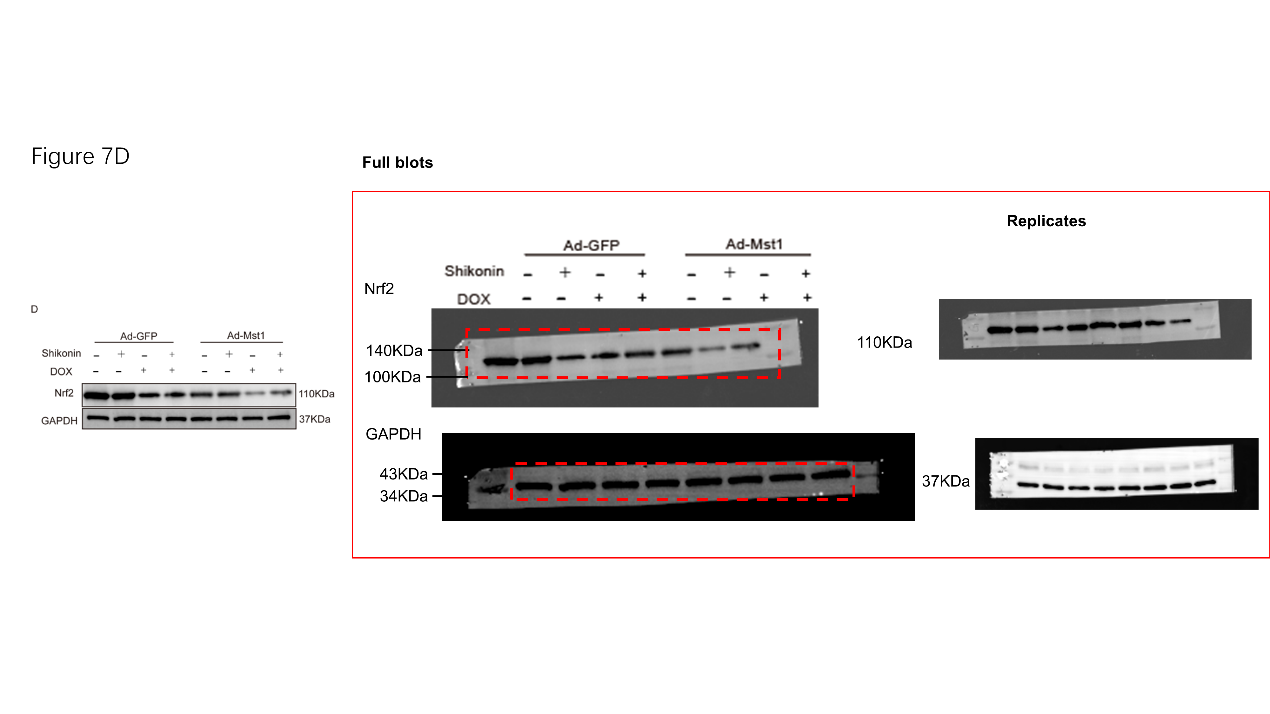


Figure S9. Original blots/gels of Figure7D.


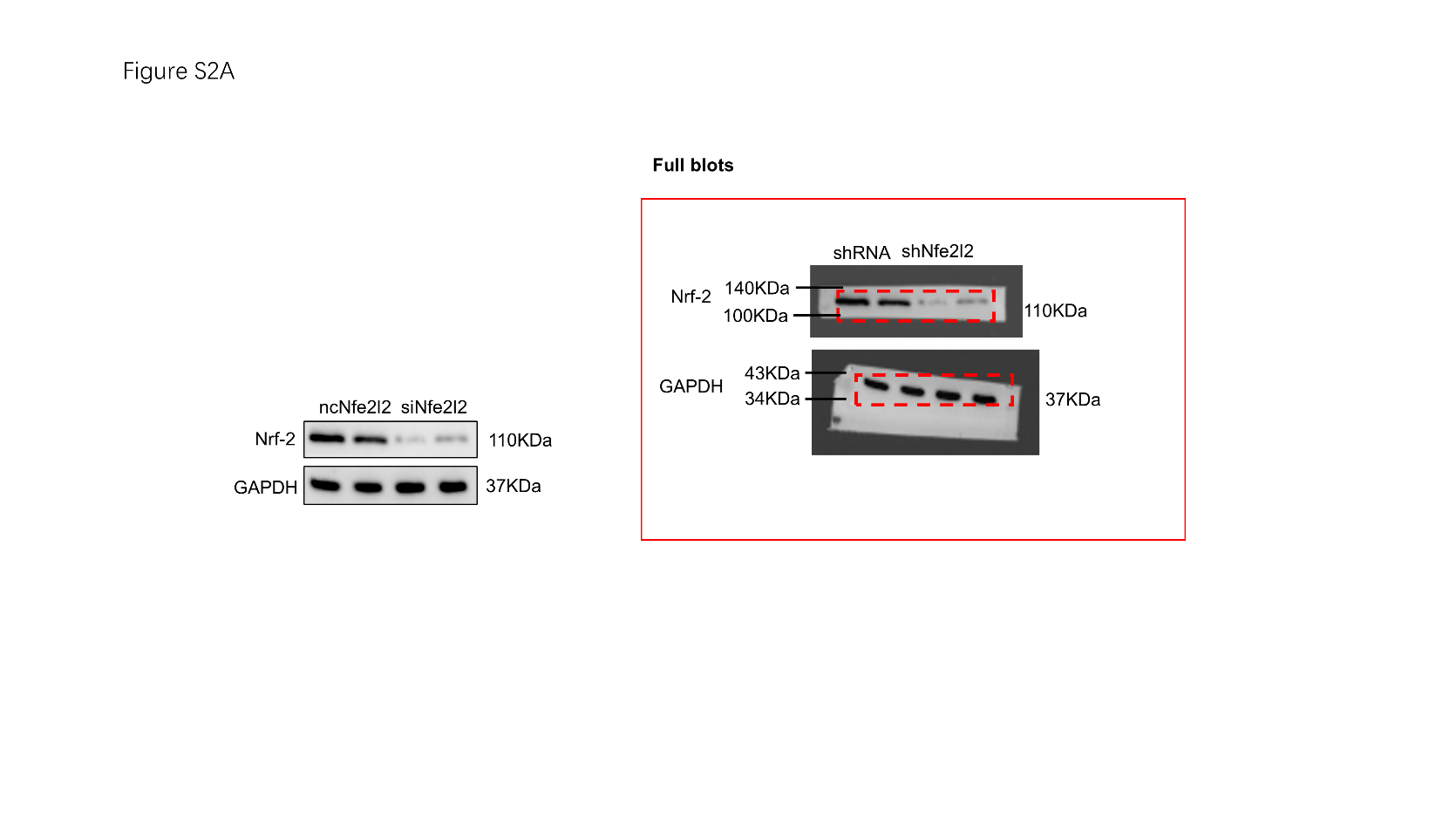


Figure S10. Original blots/gels of FigureS2A.


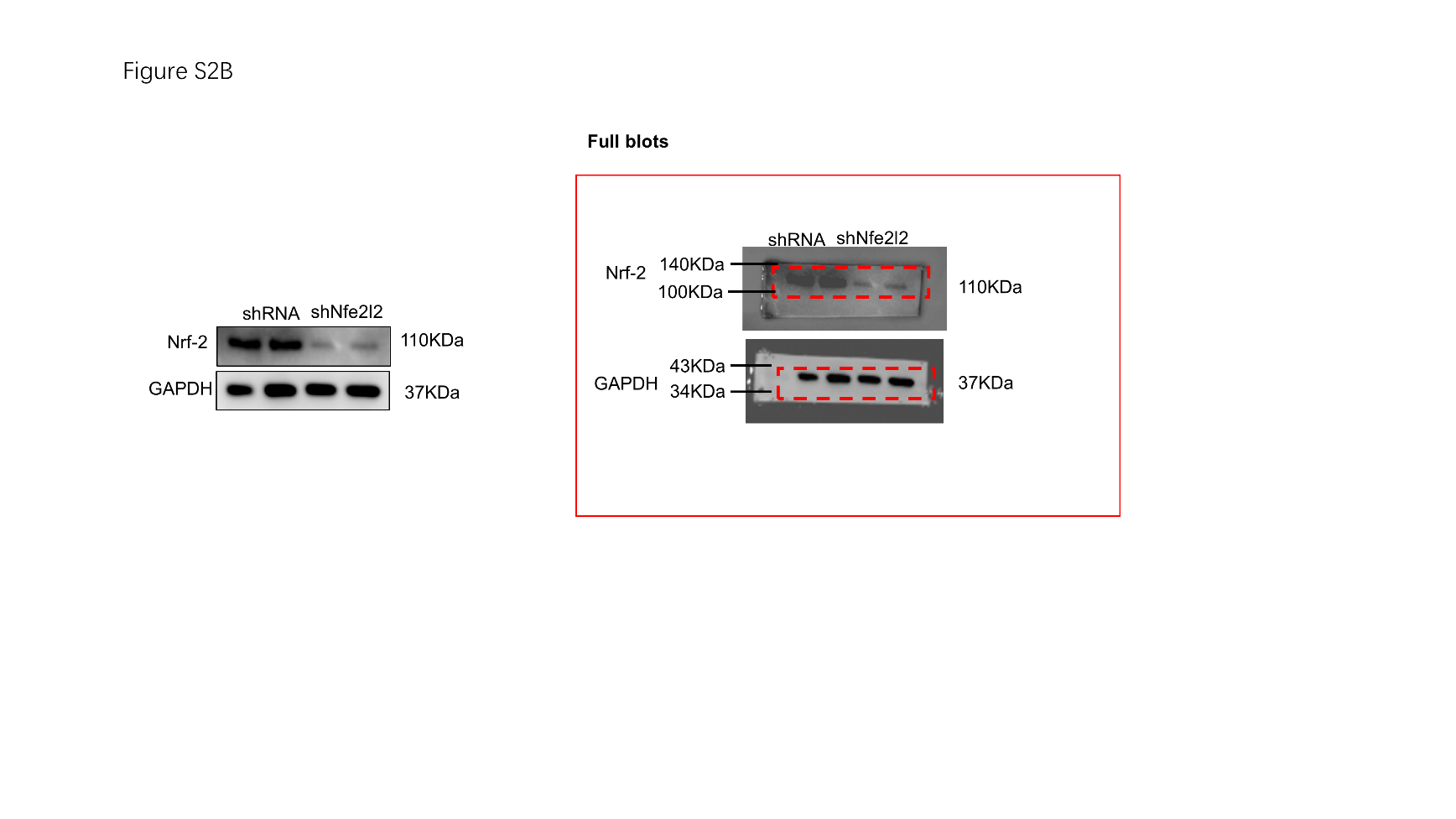


Figure S11. Original blots/gels of FigureS2B.


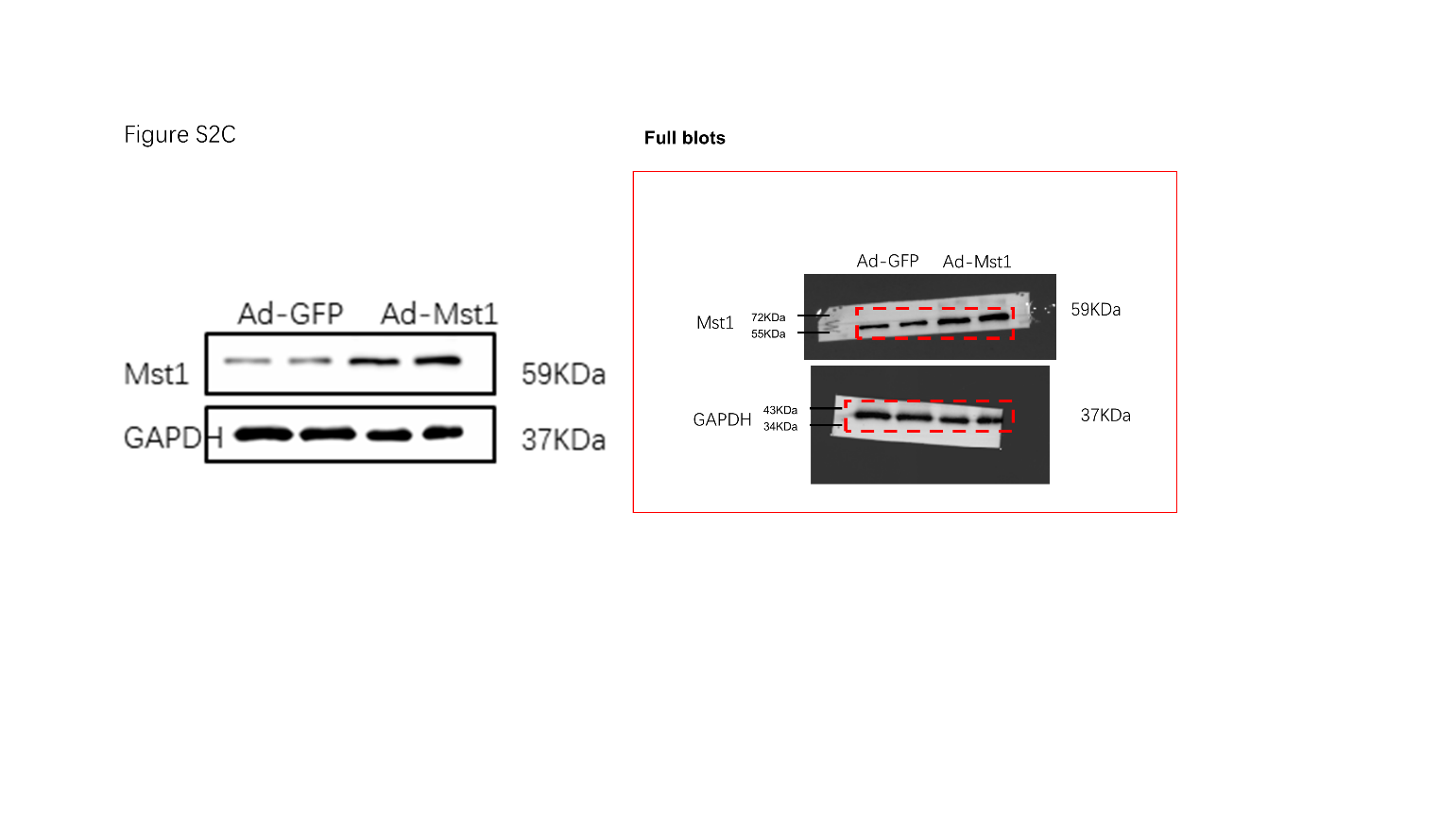


Figure S12. Original blots/gels of FigureS2C.


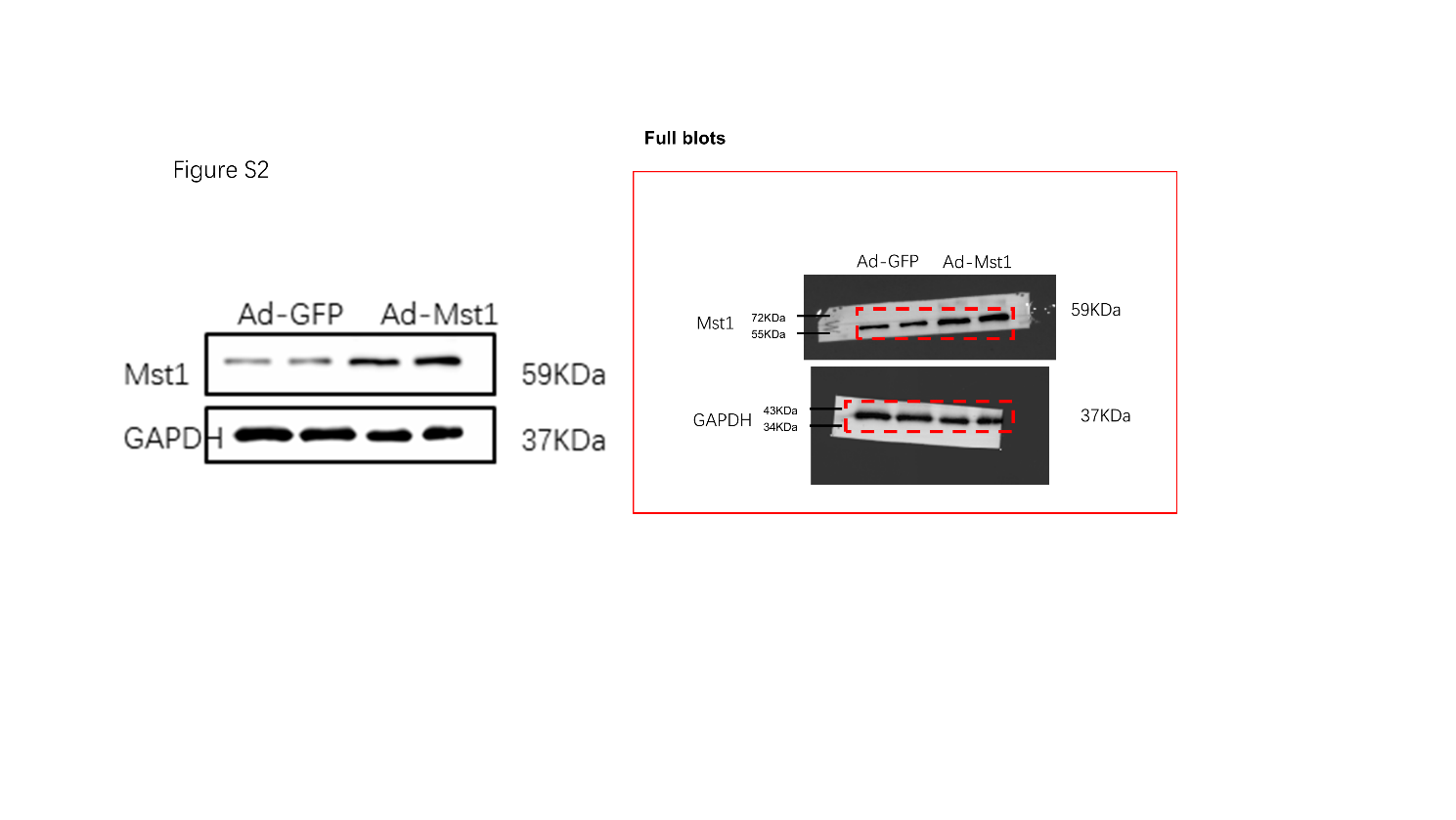


Figure S10. Original blots/gels of Figure S2C.
